# *Insights* into the Evolution of Environmental Health

**DOI:** 10.1177/117863020900300001

**Published:** 2009-03-13

**Authors:** Timothy Kelley

**Affiliations:** Environmental Health Sciences Program, Department of Health Education and Promotion, College of Health and Human Performance, East Carolina University, Greenville, NC

It is now very clear that during its short tenure of only one year, the value of the new Libertas Academia online, open access journal *Environmental Health Insights* has been demonstrated through both the authors’ contributions and readers’ responses. Just to briefly review, since it was launched in March of 2008, EHI has published 28 peer-reviewed articles on a wide variety of topics relevant to the discipline of environmental health with over 30,000 reader views. While I do not imply that quantity is necessarily an indicator of quality, this is more articles and views than all but 10 of the 86 journals listed on the Libertas Academia website (www.la-press.com), and within the top 6% most productive over time, which indicates a significant professional and public interest in environmental health issues. Quality being the more important concern, I also feel confident that *Environmental Health Insights’* Editorial Board has maintained a rigorous standard for publication, while acknowledging the need for both databased and review articles appropriate for an online open-access journal. This has been accomplished through their application of a professional, responsible and thorough peer-review process. I thank *Environmental Health Insights* Board members, contributors and our readers for their service to and interest in *Environmental Health Insights* and to the discipline of environmental health.

Not surprisingly, a majority of *Environmental Health Insights’* articles fit within the broad category of environmental health epidemiology, while other multiple articles fit within diverse categories of topics from recreation, physical activity and lifestyle to sanitation practices, and diet and health. Other individual articles focus on environmental health economics, policy, research ethics, social issues, eco-psychiatry, and climate change and health. A wide range of international contributors are also represented, including those from Australia, Canada, Finland, India, Israel, Japan, Kazakhstan, Spain, Sweden, the United Kingdom, the United States, and Zambia. A range of internationally-recognized and respected organizations are also represented, including the Agency for Toxic Substances and Disease Registry (ATSDR), the Centers for Disease Control and Prevention (CDC), the National Institute of Environmental Health Sciences (NIEHS), the United Nations Children’s Fund (UNICEF) and the World Bank. If you have not yet done so, I invite and encourage you to take time to review these articles, as I believe that their content has much to contribute to our individual and group efforts to improve environmental health around the world.

Evidence of the changing focus of environmental health is evident in the content of *Environmental Health Insights*. From more traditional epidemiology of acute exposures and infectious disease, the discipline focuses increasingly on chronic health issues and noninfectious disease related to environmental exposures. Environmental health economic, social science and policy issues are becoming much more important, including examples focusing on ethical research practices, migration, childhood environmental exposures, climate change and energy policy. It is also evident that lifestyle choices such as smoking, diet, physical activity, and recreation are becoming more important.

Having made these observations concerning *Environmental Health Insights*, I would like to reflect briefly on what I have characterized as the “evolution” of environmental health over the last 100 years.

February, 2009 is the 200th anniversary of the birth of Charles Darwin and the 150th anniversary of the publication of his “On the Origin of Species.” Many authors have previously described how environmental health has contributed to huge advancements in both the quantity and quality of human life. This has been accomplished through improved water, food, and air quality; improved waste management practices; public health advances such as immunization and antibiotics; and public health education; among many other efforts. Rather than recounting a history of environmental health, this editorial seeks to take it’s inspiration from a recent issue of *Scientific American* (“The Evolution of Evolution”, January, 2009) which seeks to extend some of the basic concepts of evolution to apply them to criminology, microbiology, medicine, molecular biology, ecology, and metagenomics. Can basic principles of evolution such as resource allocation and natural selection and more advanced concepts such as punctuated equilibrium be applied to the discipline of environmental health to better understand how the discipline has changed?

**Resource allocation** is a driving force of evolution. If a population has access to an adequate supply of resources, it encounters little pressure to change. Certainly, funding resources allocated to environmental health (and many other disciplines) have been restricted from the local to the federal level, which has encouraged environmental health professionals and researchers to make the most efficient use of funding available. Novice environmental health professionals sometimes tend to think that their job is simply to identify a problem and locate the resources to solve it. Unfortunately, we quickly learn that most of the work is in prioritizing needs and matching the funding resources available to address those needs in the most efficient way possible.

**Natural selection** is the basis of evolution. One tenant of evolution is that individuals do not evolve—populations evolve. To use a popular example, a giraffe’s neck is not long because it stretched to reach the treetops and then passed this trait on to it’s offspring, but rather because those animals with naturally longer necks had a reproductive advantage over those with shorter necks and therefore increased in the population. To apply this concept to environmental health, individual professionals may find it difficult to change practices that have been adopted by the system of which they are a part. However, when the system changes, it may be more accepting of new (potentially improved) ideas and approaches. Survival of the “fittest” doesn’t necessarily mean the “best” or most efficient, but simply what works best under a restricted set of circumstances. This change in the system could perhaps be due to a change in administrative personnel or policy or a social paradigm shift. To use an example from a previous editorial, the paradigm shift from waste management to pollution prevention demonstrates an “evolutionary” shift in environmental health from reactive to proactive management strategies. There has also been a paradigm shift on the part of the public towards understanding the health implications of their environmental exposures, including personal health choices.

**Punctuated equilibrium** is a theory developed by paleontologists Niles Eldredge and Stephen Jay Gould and to explain why significant evolutionary changes seem to occur over a relatively short period of time while much longer periods of time may result in little or no evident change. Selective pressure plays a major part in this theory, since it’s previously been acknowledged that its absence may result in little or no evident change. However, when selective pressure becomes significant, populations must change—or they perish. In addition to resource (funding) allocation, other pressures on environmental health professionals are politics, public perception, demographics, and many others. These changes are often not incremental and steady—significant changes may occur and responses developed quickly. Improved sanitation, the development of antibiotics and vaccines and increased emphasis on occupational health resulted in rapid improvements in environmental health during the 20th century. The focus has now shifted to the much slower, and perhaps more frustrating, process of identifying and controlling a variety of environmental exposures and personal lifestyle choices that may yield negative health consequences.

However and whenever they are initiated, when pressures occur, changes are also more likely to occur. Sometimes these changes are for the better, while other times they may perhaps be seen as simply responding to external pressure as a purely reactionary manner, or simply due to random chance. When random changes occur, those that confer an advantage are more likely to be retained. As in evolution, environmental health improvements tend to become a more permanent part of the system, while reactionary or simply random changes tend to be dropped due to their ineffectiveness or inefficiency. Environmental health is clearly in the process of “evolving” to better address the issues and concerns of human interactions with their environment. I am pleased to observe that *Environmental Health Insights* is both reflecting and contributing to this important process.

